# High Precision Speech Keyword Spotting Based on Binary Deep Neural Network in FPGA

**DOI:** 10.3390/e27111143

**Published:** 2025-11-07

**Authors:** Ang Zhang, Jialiang Shi, Hui Qian, Junjie Wang

**Affiliations:** College of Physics and Information Engineering, Fuzhou University, Fuzhou 350108, China; zhangang@fzu.edu.cn (A.Z.); sjl1207934463@163.com (J.S.); 112200222@fzu.edu.cn (J.W.)

**Keywords:** keyword spotting, binary neural network, deep learning, FPGA, information theory

## Abstract

Deep Neural Networks (DNNs) are the primary approach for enhancing the real-time performance and accuracy of Keyword Spotting (KWS) systems in speech processing. However, the exceptional performance of DNN-KWS faces significant challenges related to computational intensity and storage requirements, severely limiting its deployment on resource-constrained Internet of Things (IoT) edge devices. Researchers have sought to mitigate these demands by employing Binary Neural Networks (BNNs) through single-bit quantization, albeit at the cost of reduced recognition accuracy. From an information-theoretic perspective, binarization, as a form of lossy compression, increases the uncertainty (Shannon entropy) in the model’s output, contributing to the accuracy degradation. Unfortunately, even a slight accuracy degradation can trigger frequent false wake-ups in the KWS module, leading to substantial energy consumption in IoT devices. To address this issue, this paper proposes a novel Probability Smoothing Enhanced Binarized Neural Network (PSE-BNN) model that achieves a balance between computational complexity and accuracy, enabling efficient deployment on an FPGA platform. The PSE-BNN comprises two components: a preliminary recognition extraction module for extracting initial KWS features, and a result recognition module that leverages temporal correlation to denoise and enhance the quantized model’s features, thereby improving overall recognition accuracy by reducing the conditional entropy of the output distribution. Experimental results demonstrate that the PSE-BNN achieves a recognition accuracy of 97.29% on the Google Speech Commands Dataset (GSCD). Furthermore, deployed on the Xilinx VC707 hardware platform, the PSE-BNN utilizes only 1939 Look-Up Tables (LUTs), 832 Flip-Flops (FFs), and 234 Kb of storage. Compared to state-of-the-art BNN-KWS designs, the proposed method improves accuracy by 1.93% while reducing hardware resource usage by nearly 65%. The smoothing filter effectively suppresses noise-induced entropy, enhancing the signal-to-noise ratio (SNR) in the information transmission path. This demonstrates the significant potential of the PSE-BNN-FPGA design for resource-constrained edge IoT devices.

## 1. Introduction

In recent years, the rapid advancement of the Internet of Things (IoT) and Artificial Intelligence (AI) technologies has significantly promoted the widespread adoption of voice-enabled AI assistants in various IoT terminals, such as smart homes and wearable devices [1]. To further enhance the battery life of portable IoT terminals, Keyword Spotting (KWS) has emerged as a critical technology. Serving as the intelligent wake-up engine for AI assistants, KWS ensures that IoT terminals can intelligently switch between sleep and wake states during user operation intervals, making it an indispensable component [2].

As KWS operates continuously, its power consumption constitutes a major portion of the energy expenditure of AI assistants and the entire IoT terminal [2]. Achieving high recognition accuracy while effectively reducing KWS power consumption is a significant challenge actively explored by both academia and industry. Early KWS systems primarily relied on traditional machine learning methods, such as Gaussian Mixture Models (GMMs) and Hidden Markov Models (HMMs), for speech recognition. These methods are susceptible to interference from noise, accent variations, or speaker changes, resulting in relatively low recognition rates. Recently, with the rise of Deep Neural Networks (DNNs), DNN techniques with superior recognition capabilities have become the mainstream trend in the KWS field [3]. However, DNNs are inherently computation- and memory-intensive. Deploying DNN-KWS modules on hardware resource-constrained portable IoT terminals is an extremely challenging task [4]. This challenge aligns with the core trade-off in Rate-Distortion Theory, where one seeks the minimal bit-rate (model size/complexity) for a given level of accuracy (distortion).

Network quantization is an effective method for compressing DNN models and addressing deployment difficulties [5]. It can be viewed as an efficient encoding strategy guided by information theory, representing network parameters and activations with minimal bits to reduce information loss. M. Shah et al. [6] first proposed a quantized three-layer Convolutional Neural Network (CNN) design for KWS tasks. Compared with a floating-point three-layer CNN, the quantized CNN achieved 89.5% recognition accuracy using only 6-bit weights and 16-bit fixed-point computations, consuming merely 14% of the power of the full-precision model. This work strongly demonstrates the potential of quantization in reducing DNN-KWS power consumption.

Given that binary network quantization can simplify the complex multiply–accumulate (MAC) operations in convolution into efficient XNOR logic operations, thereby maximizing the reduction in computational complexity and memory resource consumption in DNN-KWS, Binary Neural Networks (BNNs) have become a focal point for researchers in the DNN-KWS field [7]. Binarization can be conceptualized as transmitting original high-precision information through an extremely low-capacity channel. The core challenge is to maintain sufficiently high mutual information between the input features and the final decision under this constraint. Zheng et al. [8] proposed a BNN-KWS design based on a two-layer fully connected network. This design reduced the computational load of the KWS module by 94% through network binarization while maintaining 91% recognition accuracy. To further reduce the number of XNOR logic units in BNN-KWS, Liu et al. [9] introduced approximate computing into the BNN-KWS design. Their design incorporated a Signal-to-Noise Ratio (SNR) prediction module, enabling adaptive dual-mode configuration of standard and approximate computation, thereby reducing the computational complexity of BNN-KWS to approximately 1.2% of the full-precision model. However, the approximate computing strategy caused the recognition accuracy of BNN-KWS to drop to 87.9%. To improve the computational precision of BNN-KWS, Yu Gong et al. [10] introduced a quality assessment mechanism into the approximate computing BNN-KWS design, achieving 88.1% recognition accuracy with only 1.6% of the computational complexity of the full-precision model. Subsequently, Lin et al. [11] increased the KWS recognition rate to 95% by appropriately increasing the network depth.

These network binarization methods effectively reduce DNN-KWS power consumption by lowering the computational complexity of the DNN module. However, since both parameters and activation functions in binarized networks are represented by only 1 bit, the model’s expressive power is limited, and optimization is challenging [12]. Consequently, the recognition rates of most existing BNN-KWS modules do not exceed 95% [13]. From an information theory standpoint, the 1-bit representation severely constrains the amount of information that can be transmitted per layer, potentially creating an information bottleneck during the forward pass. This leads to the loss of critical information and often results in output probability distributions with high Shannon entropy, indicating significant uncertainty. In practical applications, KWS modules with lower recognition rates experience frequent false wake-ups, increasing the average power consumption of AI assistants [13]. To solve this problem, this paper proposes a speech-enhanced BNN model, namely the Probability Smoothing Enhanced Binarized Neural Network for KWS (PSE-BNN), which improves BNN-KWS recognition accuracy by employing smoothing filtering to reduce noise during the recognition process. The design is inspired by the information processing principle of utilizing temporal correlation to reduce uncertainty. The main innovative contributions of this work are as follows:

- Probability Smoothing Enhanced Binarized Neural Network (PSE-BNN): We propose a novel PSE-BNN model featuring a two-layer hierarchical architecture. The first layer takes Mel-Frequency Cepstral Coefficients (MFCCs) as inputs to extract preliminary probability features. The second layer leverages temporal correlations between consecutive speech frames to apply smoothing filtering to the outputs of the first layer, acting as an SNR enhancer by utilizing the mutual information between adjacent frames to reduce the conditional entropy of the current frame’s output, enhancing keyword recognition accuracy. Evaluated on the open-source Google Speech Commands Dataset (GSCD) [14], the PSE-BNN achieves a recognition accuracy of 97.29%. The entropy of the output probability distribution is significantly reduced after smoothing.
- FPGA-Based Hardware Implementation: We designed and implemented an efficient hardware circuit for the PSE-BNN model on an FPGA. Optimizations include employing approximate computing and Coordinate Rotation Digital Computer (CORDIC) algorithms to replace MAC operations in the network’s logic operations and the newly added smoothing filter layer with logical computations. Implemented on the Xilinx VC707 development board (Xilinx, Inc., San Jose, CA, USA), the PSE-BNN module occupies only 0.64% of LUTs, 0.14% of FFs, and utilizes no DSP resources. This design achieves efficient low-power information processing, aligning with the fundamental principles of information theory concerning energy, computation, and information.
- Comprehensive Evaluation: We present detailed software and hardware-in-the-loop (HIL) test results, demonstrating the model’s high accuracy and low resource utilization, significantly outperforming state-of-the-art designs in both metrics (accuracy +1.93%, resources −65%).

## 2. BNN-KWS System Based on MFCC

### 2.1. System Architecture

**Figure 1:**
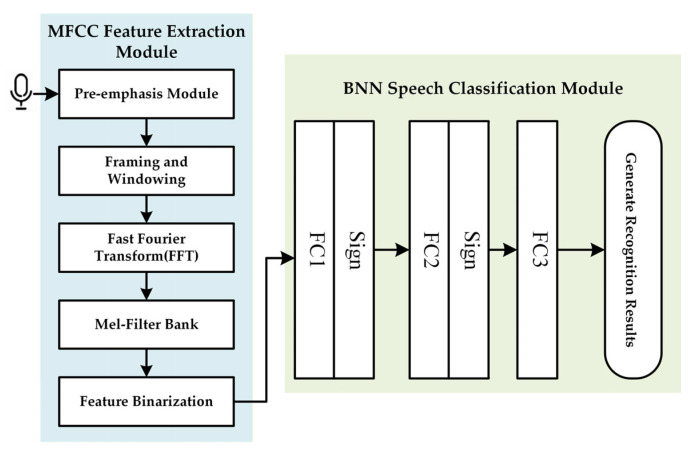
Structure of a typical KWS system.

As illustrated in Figure 1, a typical BNN-KWS system consists of two main modules: the MFCC feature extraction module and the BNN-based speech classification module.

#### 2.1.1. MFCC Feature Extraction Module

The MFCC feature extraction module comprises five stages:

- **Pre-emphasis Module**: A first-order high-pass filter $H\left(z\right)=1-az^{-1}$ with a pre-emphasis coefficient a (typically between 0.9 and 1.0) is applied to attenuate high-frequency noise.
- **Framing and Windowing**: The speech signal is segmented into data blocks of length N using a window function $W\left(n\right)$. A Hamming window of length N = 256 is generally employed.
- **Fast Fourier Transform (FFT)**: A 256-point FFT converts the segmented time-domain speech signal into the frequency domain.
- **Mel-Filter Bank**: An 11th-order triangular bandpass filter bank processes the frequency-domain signal to enhance formants and extract spectral features.
- **Feature Binarization**: A threshold of 0.4 is applied to quantize spectral features into binary values. This binarization process is a form of information compression, aiming to retain the most critical information for classification, akin to feature selection or source coding in information theory.

#### 2.1.2. BNN Speech Classification Module

The speech classification module is the core of the KWS system and primarily consists of a binarized neural network. The task of this module is to extract the maximum information relevant to the keyword from the compressed binary features, i.e., to maximize the mutual information between the features and the class labels. Based on [6], we adopt a three-layer fully connected network suitable for edge devices and apply binary quantization to obtain the final BNN model.

The current edge-based KWS demonstrates enhanced accuracy when augmented with a fully connected layer. Accordingly, this study designs a three-layer fully connected network optimized for edge devices and applies binary quantization to derive the final BNN model.

### 2.2. Analysis of Weight Parameters and Recognition Accuracy for BNN Modules

Using the GSCD as the test dataset, we compared the parameter count and recognition accuracy between the original CNN and the quantized BNN. Table 1 shows that binary quantization reduces the network parameter size to only 3.1% of the original CNN module’s total parameters. However, this reduction comes at the cost of a significant decrease in recognition accuracy. This phenomenon aligns with the fundamental trade-off in Rate-Distortion Theory: a lower bitrate (smaller model size) typically results in higher distortion (lower accuracy). Since lower recognition accuracy increases the frequency of false wake-ups in KWS, designing hardware-oriented KWS speech recognition modules requires careful balancing of weight parameters and recognition rates.

## 3. PSE-BNN Model for KWS

### 3.1. Network Architecture

**Figure 2:**
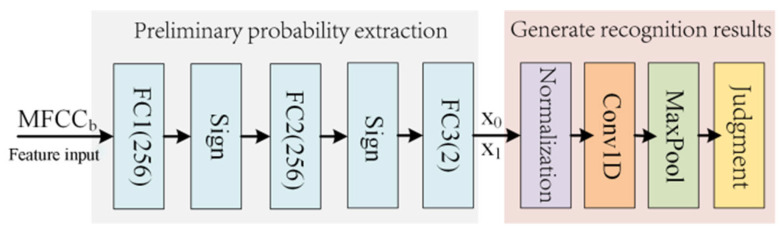
Proposed PSE-BNN model.

To address the insufficient recognition accuracy of existing BNN models in KWS designs, this paper proposes an enhanced BNN module. Figure 2 illustrates the proposed PSE-BNN model. The PSE-BNN consists of two components: the Preliminary Probability Extraction (PPE) layer and the Recognition Result Generation (RRG) layer, aiming to improve decision accuracy by increasing network depth. The design philosophy stems from hierarchical information processing theory, where preliminary information extraction is performed at shallow layers, followed by refined decoding and denoising using contextual information at deeper layers, thereby reducing the uncertainty of the final decision.

#### 3.1.1. Preliminary Probability Extraction (PPE) Layer

The PPE layer primarily comprises two fully connected (FC) layers (FC1 and FC2) with 256 neurons each, one FC layer (FC3) with 2 neurons, and two Sign activation function layers. Due to binary quantization, the multiplication operations in the FC layers of the PSE-BNN are implemented using simple XNOR bitwise operations. Therefore, the computation for layers FC1 and FC2 can be expressed as:

(1) $$z^{i}=\sum_{j=1}^{256}w_{j}^{i}⊙x_{j}^{i}-b^{i}$$

where i is the index of the FC layer, $x_{j}^{i}$ is the single-bit input data of the FCi layer, $w_{j}^{i}$ represents the corresponding single-bit weight, $b^{i}$ denotes the bias term, and $z^{i}$ is the output of the FCi layer. The subtraction of the bias term is employed to adjust the activation threshold of neurons, ensuring the accuracy of binarized activation. In the FPGA-based hardware implementation, the bias subtraction is integrated with XNOR and accumulation operations, realized via a streamlined arithmetic logic unit (ALU), thereby providing stable input for subsequent smoothing filter and decision-making layers. The network activation layer utilizes the *Sign* function to map continuous output values to two discrete values, +1 and −1. Specifically, this can be expressed as:

(2) $$z_{binary}^{i}=Sign(z^{i})=\left\{\begin{array}{c} +1,\;z^{i}\geq 0\\ -1,\;z^{i}<0 \end{array}\right.$$

where $z_{binary}^{i}$ is the quantized activation value.

In the KWS system, the computations of FC1 and FC2 layers can be combined with the activation layer to optimize the PSE-BNN model by setting the bias term $b^{i}$ appropriately. By combining Equations (1) and (2), the binarized output values of FC1 and FC2 can be obtained as:

(3) $$z_{binary}^{i}=Sign(z^{i})=\left\{\begin{array}{c} +1,\;\sum_{j=1}^{256}w_{j}^{i}⊙x_{j}^{i}\geq b^{i}\\ -1,\;\sum_{j=1}^{256}w_{j}^{i}⊙x_{j}^{i}<b^{i} \end{array}\right.$$

The FC3 layer generates the raw output $z^{3}$ without binarization.

After processing through FC1, FC2, FC3, and the *Sign* function, the Preliminary Probability Extraction (PPE) layer outputs the preliminary prediction probabilities for both “keyword class” and “non-keyword class” of the voice signal. These preliminary probabilities reflect, to some extent, the presence of keywords in the speech, providing important basis for subsequent processing. However, due to the truncation errors introduced by binary quantization, these output probabilities are not stable, which directly affects the decision-making process. Therefore, this paper adds a Recognize Results Generating (RRG) layer.

#### 3.1.2. Recognize Results Generating Layer

The Recognize Results Generating (RRG) layer consists of a normalization layer, a smoothing filter convolutional layer, a pooling layer, and a decision-making layer. The core function of this layer is to filter the preliminary probability sequence using the temporal correlation of speech signals (i.e., the high mutual information between frames), reducing the entropy increase caused by noise and enhancing the SNR of useful information. To reduce the computational requirements of the model, this paper employs the *ReLU* function as the normalization function. Specifically:

(4) Pi=ReLU(zj3)∑i=01ReLU(zj3)

where $z_{i}$ is the preliminary probability, and $P_{i}$ is the optimized normalization result. Considering the correlation between speech signals, this paper employs a one-dimensional convolutional layer (Conv1D) with a length of 8 to process the probability data of several frames before and after. The convolutional kernel weights can be interpreted as estimates of the importance of information at different time points. The filtering process is a form of linear encoding of the time-series information, aiming to preserve trend information and filter out burst noise (high entropy perturbations). The basic operation of the convolutional layer can be expressed as:

(5) $${P_{j}}^{'}=\sum_{k=j-3}^{j+4}W_{k}\times P_{k}$$

where $W_{k}$ is the filter weight factor, $P_{k}$ is the input probability, and ${P_{j}}^{'}$ is the output probability. Although smoothing filtering can reduce noise spikes and false alarm rates, it may increase the probability of missing keyword edges. Therefore, this paper adds a Maxpool layer after smoothing filtering. This layer has a length of 4 and a sliding stride of 1. The calculation formula is as follows:

(6) $$C_{j}=\underset{j-1\leq k\leq j+2}{max}P_{k}^{'}$$

where $C_{j}$ is the confidence score output by the pooling layer. Max pooling here serves to further reduce uncertainty by selecting the most informative response; the entropy of its output is typically lower than that of its input. The final decision-making layer determines the final output by comparing the confidence scores of the keyword and non-keyword categories. Specifically, this can be expressed as:

(7) $$result=\left\{\begin{array}{c} 1,\;C_{keyword}\geq C_{filter}\\ 0,\;C_{keyword}<C_{filter} \end{array}\right.$$

The entire RRG layer can be viewed as a sequential information processor whose goal is to maximize the mutual information between the output and the true label while minimizing irrelevant information (i.e., noise entropy) introduced by noise.

### 3.2. Loss Function

During the training process, this paper primarily utilizes the binary cross-entropy loss function to train and update the parameters of the PSE-BNN model through gradient descent methods. The cross-entropy loss function measures the Kullback–Leibler (KL) Divergence between the true distribution (labels) and the predicted distribution. Minimizing the cross-entropy is equivalent to minimizing the information difference between these two distributions, thereby driving the model’s predictive distribution closer to the true distribution. The binary cross-entropy loss function can be specifically expressed as:

(8) $$L=\sum_{i=1}^{2}L_{i}=y_{i}\log(p_{i})+(1-y_{i})\log(1-p_{i})$$

where $y_{i}$ is the sample label, $p_{i}$ is the predicted probability, and *L* is the loss value.

The entire PSE-BNN model is trained end-to-end, jointly optimizing both the Preliminary Probability Extraction (PPE) and Recognition Result Generation (RRG) layers. Training employs the binary cross-entropy loss (Equation (8)) on the Google Speech Commands Dataset (GSCD), with all parameters—including binarized weights and RRG filter coefficients—updated via backpropagation. This data-driven approach ensures the smoothing filter is learned directly, reducing output uncertainty and enhancing recognition confidence. The bit-width values and filter parameters were also refined during retraining to ensure consistency with the FPGA implementation.

## 4. FPGA-Based Hardware Implementation of PSE-BNN

**Figure 3:**
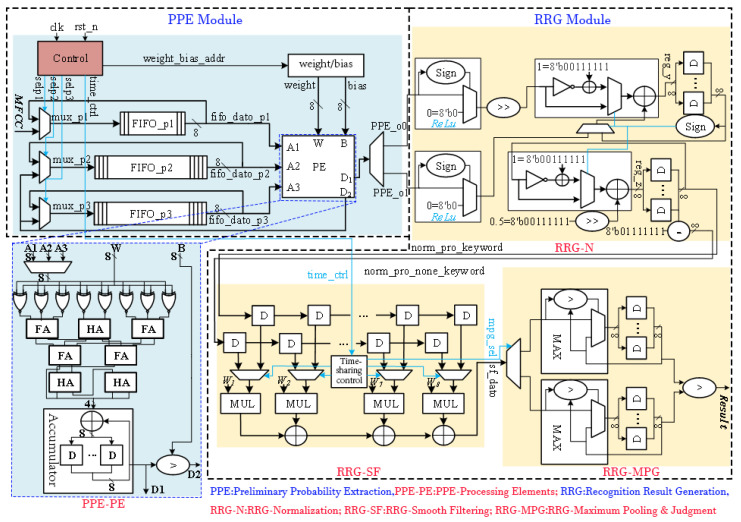
The overall circuit architecture of PSE-BNN.

This section presents the hardware design of the proposed PSE-BNN model, with the specific circuit architecture shown in Figure 3. The goal of the hardware implementation is to physically realize the aforementioned information processing flow efficiently, ensuring reliable processing of the information stream under low-power and low-latency constraints.

### 4.1. PPE Module Implementation

The PPE module, receiving MFCC data and generating preliminary probabilities, handles the computations of the Preliminary Probability Extraction layer. A circular buffer module caches input data and intermediate data from the FC1 and FC2 layers. This buffer module is pivotal for enabling the sequential reuse of a single Processing Element (PE) across all three FC layers. By temporarily storing the intermediate results, the buffer provides the necessary data interface for the time-multiplexed execution of FC1, FC2, and FC3 on the same physical hardware. By reusing Processing Elements (PEs), the module performs inference calculations for FC1, FC2, FC3, and the intermediate *Sign* layers. This reuse strategy exemplifies resource-optimized allocation in information processing, using limited hardware resources to handle a defined information processing task.

The circular buffer module consists of one FIFO with a depth of 50 and two FIFOs with depths of 256, caching input data and the FC1/FC2 intermediate data. A multiplexer (MUX) controls data reads and writes. While a single PSE-BNN computation is ongoing, the FIFOs repeatedly read and write their own output data. Upon completion, new audio feature values are loaded into the FIFOs.

The PE unit primarily performs the accumulation and comparison operations described by Equation (3), as shown in the circuit structure within Figure 3. Input signals A1, A2, and A3 correspond to the audio feature input vector and intermediate data vectors from FC1 and FC2 layers, respectively. W and B are the input weight vector and bias term. Output signals include the raw output D_1_ from FC3 and the binary activation output D_2_, reused for FC1/FC2 layers. A Control module manages a MUX to route D_2_ to the appropriate FIFO. Within the PE: (1) An array of 8 XNOR gates performs single-bit vector multiplication between inputs and weights; (2) A Wallace tree adder (composed of 3 half-adders and 4 full-adders) sums the product vectors; (3) An accumulator sums multiple groups of these sums to yield the convolution result D_1_; (4) A numerical comparator compares D_1_ with the bias B to generate the single-bit output D_2_. The XNOR parallel array enables high-speed, low-power forward propagation of information.

### 4.2. RRG Module Implementation

The RRG module implements the Recognition Result Generation layer (Figure 2), comprising three submodules: Normalization (RRG-N), Smooth Filtering (RRG-SF), and Maximum Pooling and Judgment (RRG-MPG). These three submodules work together to perform information refinement on the preliminary probability sequence.

The RRG-N submodule first performs *ReLU* transformation through sign bit determination and multiplexer circuits, where negative inputs are set to 0 and non-negative inputs remain unchanged. Subsequently, normalization is applied to the output. Since the PSE-BNN classification has two categories, the normalization probability can be achieved through a single division operation. Normalization ensures the legality of the probability values (summing to 1), allowing for valid entropy calculation. In the circuit design, this division is implemented using an 8-iteration Cordic algorithm. The iterative formula for the nth Cordic iteration is as follows:

(9) $$\left\{\begin{array}{c} y_{n+1}=y_{n}-\mu_{n}(0.5{)}^{n}x_{0}\\ z_{n+1}=z_{n}+\mu_{n}(0.5{)}^{n}\\ \mu_{n}=Sign(y_{n}) \end{array}\right.,0\leq n\leq 7$$

In the formula, $y_{0}$ represents the dividend, $x_{0}$ denotes the divisor, $\mu_{n}$ is the directional control factor, and the iteration result $z_{8}$ constitutes the quotient. In our design, the Cordic division circuit achieves 2× numerical scaling through shifters, implements circuit resource sharing between addition and subtraction operations using configurable adder-subtractor units, and determines specific arithmetic operations through control signals. Notably, subtraction is realized by adding the two’s complement of the input. After 8 iterative computations, the optimized normalization process described in Equation (4) completes, yielding outputs $P_{1}$ and $P_{2}$.

In the RRG-SF submodule, this design employs two shift registers to store 16 independent normalized probabilities (8 consecutive frames for both keyword and non-keyword categories). The shift registers form a short-term memory unit, preserving the temporal context of information. The convolution calculation between probabilities and weight vectors is implemented through multiply–accumulate (MAC) circuits. By utilizing multiplexers to reuse partial computation circuits, time-division processing for dual-category probability filtering is achieved, ultimately implementing the smoothing filter computation defined in Equation (5).

The RRG-MPG submodule acquires the maximum value of smoothed probabilities across 4 consecutive frames through a maximum value extraction circuit, then stores this result using register arrays, thereby implementing the confidence score computation specified in Equation (6). Finally, a decision comparator performs comparative analysis between keyword and non-keyword confidence scores to generate the final keyword recognition output (Equation (7)).

### 4.3. Timing Characteristics of the PSE-BNN Circuit System

**Figure 4:**
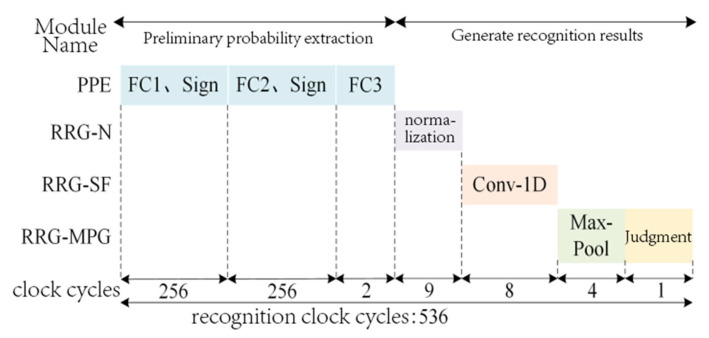
Pipelining in PSE-BNN system timing.

The timing diagram of the proposed PSE-BNN circuit system is illustrated in Figure 4. The system operates in a sequential execution mode rather than a pipelined one. This design choice is a direct consequence of the hardware-reuse strategy employed in the PPE module, where a single Processing Element (PE) is time-multiplexed across the FC1, FC2, and FC3 layers. Consequently, the system must complete the processing of one entire speech frame before commencing computation on the next. The PPE module first requires 514 clock cycles to complete the preliminary probability extraction for a single speech frame. Subsequently, the RRG module performs its normalization, smoothing, and decision-making within 22 clock cycles. Thus, the complete keyword recognition functionality for one frame totals 536 clock cycles. This fixed latency defines the total processing time from information input to decision output. While this non-pipelined approach does not support the concurrent processing of multiple consecutive frames, it achieves the design goal of ultra-low resource utilization. The resulting latency is fully sufficient for real-time KWS applications, as the processing time per frame remains significantly shorter than the typical duration of a speech frame itself, ensuring no data backlog occurs during continuous operation.

It is noteworthy that although the RRG module introduces additional processing steps, it completes its operations in merely 22 clock cycles, accounting for only approximately 4% of the total latency. This demonstrates that the proposed smoothing mechanism delivers a significant improvement in accuracy while introducing negligible latency overhead.

## 5. Experiments and Results Analysis

To verify the effectiveness of the proposed PSE-BNN model, we conducted performance evaluations using the GSCD as the test benchmark.

First, the recognition accuracy of the PSE-BNN model was validated through co-simulation of software and hardware implementations. The software testing was performed on a PC configured with the following specifications:

CPU: Intel^®^ Core™ i7-11800H

RAM: 16 GB

GPU: NVIDIA GeForce GTX 3050 (8 GB VRAM)

The software model was implemented and trained using a custom framework built in Python 3.7.

**Figure 5:**
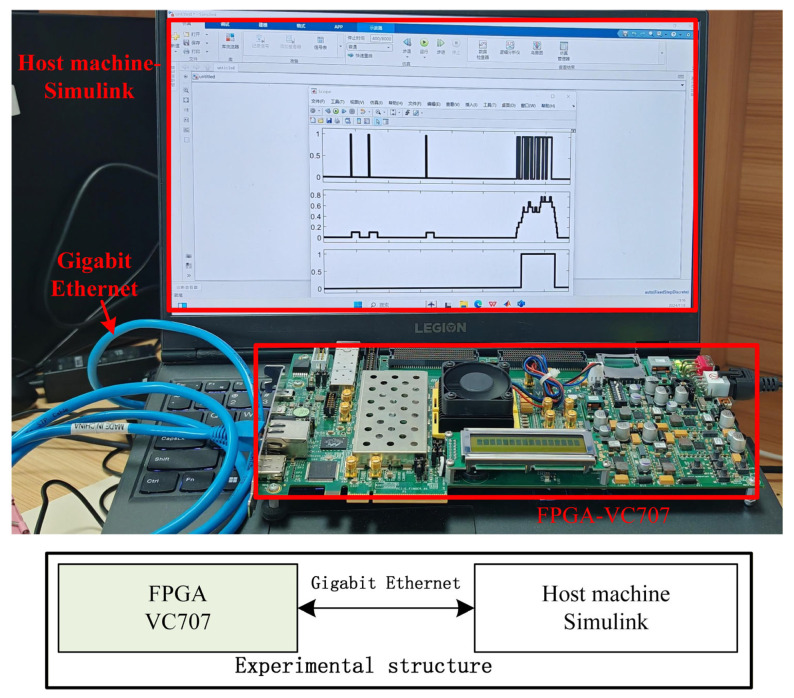
Hardware-in-the-Loop (HIL) test platform.

For hardware validation, a Hardware-in-the-Loop (HIL) test platform was implemented, as illustrated in Figure 5. The platform comprises a Xilinx FPGA VC707 development board interfaced with a host PC via Gigabit Ethernet. The PSE-BNN model was deployed on the VC707 FPGA, and the HIL platform was utilized to evaluate the hardware recognition performance of the keyword spotting (KWS) system. Given the low resource utilization of the proposed PSE-BNN on the Virtex-7 FPGA, the design is also suitable for deployment on smaller FPGAs, which are more cost-effective for edge devices.

Subsequently, this work analyzes the hardware implementation performance of the proposed KWS system. The hardware resource utilization of the KWS system is evaluated and compared with existing hardware designs.

### 5.1. Network Recognition Performance Analysis

#### 5.1.1. Software Testing Performance

In the experiments, we first converted raw audio from the GSCD into binary MFCC features using the VoiceBox speech processing toolbox in MATLAB. MFCC feature extraction itself is a compression and reshaping of speech spectral information, aiming to preserve the information most important for human hearing or for the classifier. These features were subsequently fed into the PSE-BNN model for processing.

A multi-group experimental strategy was adopted: each of the 30 keywords in the GSCD served sequentially as the positive class, while the remaining keywords and noise samples constituted the negative class. Thirty independent experiments were conducted, with the mean accuracy across all groups serving as the model evaluation metric. During network training, the learning rate was set to 0.001 with 20 epochs and a batch size of 500.

**Figure 6:**
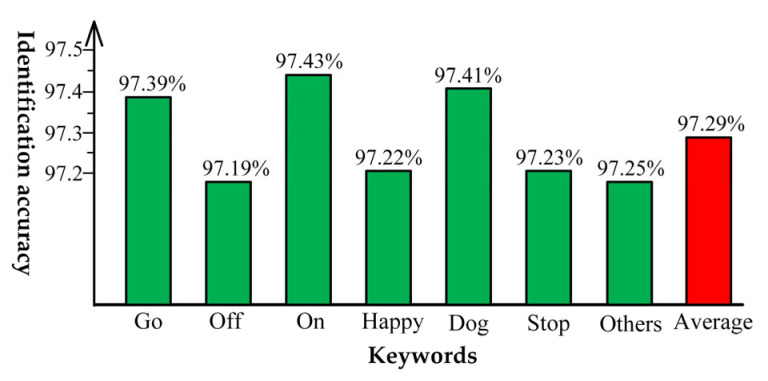
Experimental accuracy statistics.

Figure 6 presents the accuracy statistics of the PSE-BNN model on the GSCD. The results demonstrate that while recognition accuracy varies slightly across different keywords, the overall recognition accuracy reaches 97.29%.

#### 5.1.2. Hardware Testing Performance

To validate the real-time performance of the proposed PSE-BNN model, a real-time HIL test platform was established. During testing, the PC transmits binarized MFCC features to the VC707 FPGA in real-time via MATLAB/Simulink 2022b. The VC707 performs PSE-BNN hardware computations and then transmits the results back to the PC through Ethernet for visualization in MATLAB/Simulink. This closed-loop test validates the correctness and real-time performance of the complete flow of information from the software environment to hardware processing and back.

**Figure 7:**
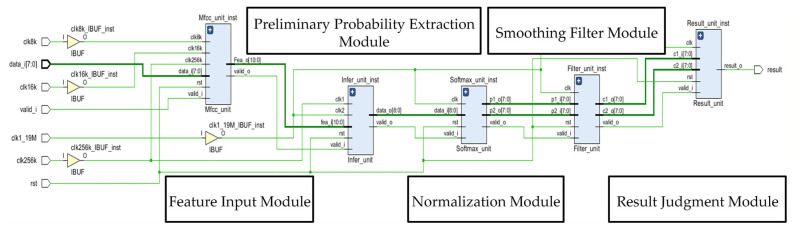
The RTL schematic diagram.

Figure 7 shows the RTL schematic diagram of the proposed system.

**Figure 8:**
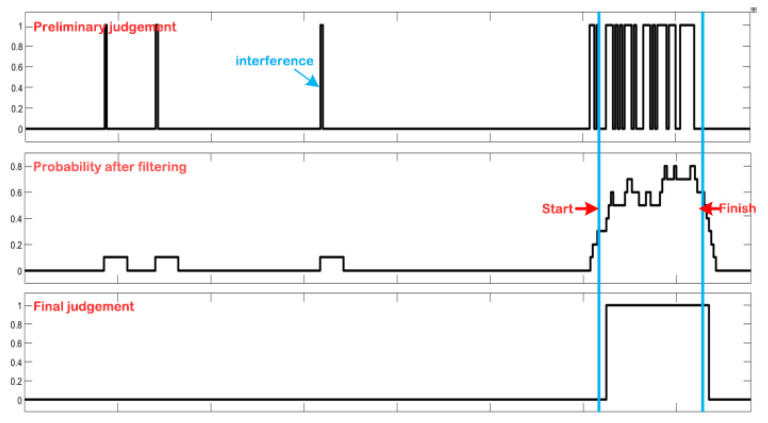
Keyword recognition output waveform.

Figure 8 Actual Processing Waveform on the HIL Platform. To validate the PSE-BNN model, the outputs of the PPE module, RRG-SF module, and RRG-MPG module in the hardware implementation are transmitted back to the PC in real-time. The waveform demonstrates that the smoothing processing effectively eliminates interference from noise and disturbance signals in continuous speech segments, thereby generating more accurate keyword recognition results. This visually demonstrates the process of information being refined layer by layer and entropy being gradually reduced.

### 5.2. Analysis of System Hardware Implementation Performance

#### 5.2.1. Hardware Resource Utilization

Table 2 details the resource utilization of the proposed PSE-BNN/KWS system. Since the convolutional operations in the PSE-BNN model involve no multiplication, the design requires no DSP resources. Furthermore, through approximation-optimized design, the implementation occupies only 1939 LUTs, corresponding to a 0.64% overall utilization rate. The single-bit characteristic of the PSE-BNN model significantly reduces memory requirements for the KWS system. By employing data concatenation storage, the design minimizes Block RAM (BRAM) usage, requiring only 6.5 BRAM blocks (equivalent to 234 Kb, assuming 36 Kb blocks). The extremely low resource utilization indicates that this design achieves a very high information processing energy efficiency ratio, i.e., the amount of information processing (or recognition performance achieved) per unit of hardware resource consumed is very high.

**Figure 9:**
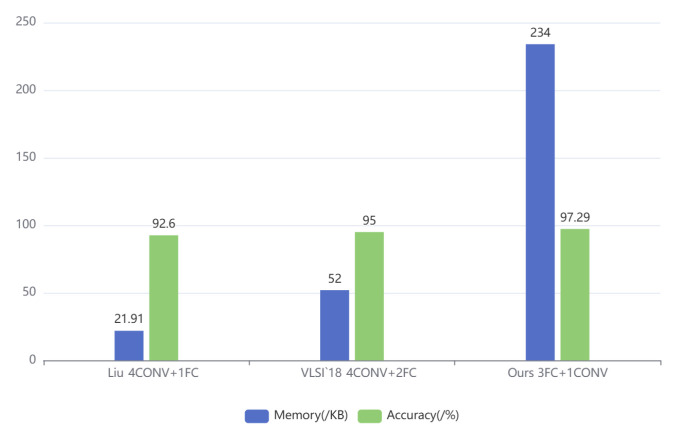
The impact of the number of FC layers on memory and accuracy [9].

Figure 9 illustrates the correlation between the number of fully connected (FC) layers and the resulting memory footprint and recognition accuracy. This trend is further corroborated by comparisons with prior implementations. For example, Liu [9] used a 4CONV + 1FC structure to achieve 92.6% accuracy with 21.91 KB memory, while the VLSI’18 [9] design, employing 4CONV + 2FC, reached 95% accuracy at 52 KB. Our architecture, which uses 3FC + 1CONV, attains a notably higher accuracy of 97.29% at 234 KB. These results confirm that augmenting the number of FC layers consistently improves accuracy, though at the cost of increased memory usage. This trade-off is intentional in our design, reflecting a deliberate choice to prioritize recognition accuracy for high-accuracy keyword spotting, even with moderate memory overhead.

#### 5.2.2. Comparison with State-of-the-Art Works and Analysis

Table 3 presents a comprehensive comparative analysis of state-of-the-art hardware designs. Most existing hardware designs primarily utilize the GSCD, either version v1 or v2, focusing on word-level decision-making for practical KWS applications at the edge. Our KWS architecture achieves a classification accuracy of 97.29%, which represents a significant improvement of 1.93 percentage points over the current state-of-the-art design [15], despite utilizing a shallower configuration (3 fully connected layers and 1 convolutional layer) compared to its 7-layer convolutional architecture. This evidence highlights the effectiveness of fully connected layers in optimizing KWS accuracy. This evidence highlights the effectiveness of fully connected layers in optimizing KWS accuracy.

Our trade-offs in the architecture become apparent when examining resource utilization. Our design increases the number of fully connected layers, necessitating a larger allo-cation of Block RAM (BRAM). To address this, we implemented layer-wise binarization of the three fully connected layers, resulting in a significant reduction in computational complexity while maintaining performance integrity. While binarization typically reduces memory, our PSE-BNN’s increased footprint stems from employing larger, more effective fully connected layers. This architectural choice, facilitated by the computational efficiency of 1-bit operations, allows us to surpass the accuracy of all prior BNN-based works [9,11,15] and even 8-bit non-BNN architectures [16,17]. Crucially, by implementing the model on an FPGA, we leverage distributed on-chip memory, which enables parallel data access and mitigates the typical latency penalty associated with larger models. When combined with pipelined/parallel logic and approximate computing, this approach not only accommodates the larger model but also enables a high throughput of 2.2 GOP/s at a low clock frequency, achieving the best performance in both accuracy and speed. In addition, to counteract any potential degradation in feature representation caused by binarization, we introduced low-bit quantized feature enhancement modules.

Furthermore, our hardware accelerator design incorporates an innovative approximate computing paradigm through algorithm-hardware co-design, which leads to a notable reduction in FPGA LUT utilization. The throughput, evaluated in Giga-Operations Per Second (GOPS), follows the methodology of [18] where each binary XOR, negation, and addition is accounted as a single operation. After a series of optimized design iterations, our implementation achieves rapid execution of KWS computations. Experimental results indicate that our design provides a throughput of 2.2 GOP/s, representing a 1.89× improvement over the current state-of-the-art solution [17] that uses GRU + FC.

Recent research has started to explore alternative neural architecture approaches for KWS system development, as seen in [16,17]. While these alternative architectures show reduced computational complexity, their reliance on high-precision arithmetic operations results in lower recognition accuracy and computational throughput compared to our implementation. These findings reinforce the conclusion that Binary Neural Networks (BNNs) continue to be the best architectural choice for edge-optimized digital KWS systems, especially when considering the trade-off between accuracy and throughput in resource-constrained environments.

Overall, the proposed PSE-BNN achieves a better trade-off, offering higher recognition accuracy and throughput while maintaining low hardware cost, making it highly suitable for resource-constrained edge KWS applications.

## 6. Conclusions

This paper presents a high-precision, high-throughput BNN-based keyword spotting (KWS) system optimized for FPGA implementation. By investigating the primary causes of limited recognition accuracy in existing BNN-KWS systems, from an information theory perspective, we identified the root cause as information loss and increased uncertainty (entropy) induced by binarization. We introduced smooth filtering into the BNN architecture, resulting in a hardware-friendly Probabilistic Smoothing Enhanced BNN (PSE-BNN) model. This model effectively reduces the entropy of the output probability distribution by utilizing temporal context information, thereby improving recognition confidence. Furthermore, the design incorporates resource-constrained optimization strategies tailored for edge-device deployment, including approximate computing and critical circuit reuse, which significantly reduce hardware complexity. Remarkably, the proposed system achieves a 2.29% improvement in recognition accuracy while operating without DSP resources and utilizing only 1939 LUTs (0.64% utilization rate). Both experimental and information-theoretic analyses demonstrate that the PSE-BNN model not only excels in traditional metrics but also shows significant advantages in the effectiveness and reliability (low entropy output) of information processing. This research provides a novel and theoretically grounded solution for achieving efficient and reliable information processing on edge computing devices with strictly constrained resources.

## Figures and Tables

**Table 1:** Parameter comparison before and after binarization.

Parameter	DNN	BNN
Weight Parameters	680 KB	21.25 KB
Recognition Rate	99.63%	93.73%

**Table 2:** FPGA resource utilization (Xilinx VC707).

Resource	Used	Available	Utilization (%)
LUT	1939	303,600	0.64
FF (Register)	832	607,200	0.14
BRAM (36 Kb)	6.5	1030	0.63
IO	15	700	2.14
BUFG (Clock)	4	32	12.50

**Table 3:** KWS system performance comparison.

Ref.	Liu [9]	Lin [11]	He [15]	Yang [16]	Chen [17]	This Work
Tech/Platform	22 nm CMOS	16 nm CMOS	Zynq-7	28 nm CMOS	65 nm CMOS	Virtex-7
Network	4CONV + 1FC	7CONV	7CONV	Skip RNN	ΔGRU + FC	3FC + 1CONV
Bit Width	1-bit	1-bit	1-bit	8-bits	8-bits	1-bit
Memory	21.91 KB	24 KB	24 KB	18 KB	24 KB	234 KB
Freq.	250 kHz	100 MHz	100 MHz	4 MHz(FE)/ 1MHz(RNN)	125 kHz	100 kHz
Throughput	0.64 GOP/s	0.7 GOP/s	0.7 GOP/s	0.07 GOP/s	1.16 GOP/s	2.2 GOP/s
Dataset	GSCDv1	GSCDv2	GSCDv2	GSCDv1	GSCDv2	GSCDv2
Accuracy	92.6%	95.0%	95.36%	92.80%	89.50%	97.29%

## Data Availability

The original contributions presented in this study are included in the article. Further inquiries can be directed to the corresponding author.
